# Baitouweng decoction modulates gut microbial production of indole-3-propionic acid and epithelial necroptosis to alleviate DSS-induced colitis in mice

**DOI:** 10.1186/s13020-025-01143-9

**Published:** 2025-07-31

**Authors:** Jingyi Hu, Hongxin Chen, Lei Zhu, Yiheng Tong, Cheng Cheng, Guoying Yan, Hong Shen

**Affiliations:** Affiliated Hospital of Nanjing University of Chinese Medicine (Jiangsu Province Hospital of Chinese Medicine), No. 155. Hanzhong Road, Nanjing, 210029 People’s Republic of China

**Keywords:** Baitouweng decoction, Ulcerative colitis, Microbial metabolism, Necroptosis, Indole-3-propionic acid

## Abstract

**Background:**

Ulcerative colitis (UC) is a kind of inflammatory disorder structuring in the colon. Baitouweng decoction (BD) derived from Treatise on Cold Damage (Shang-Han-Lun in Chinese) has been used for the treatment of UC in clinical practice for more than 2000 years. However, the clear mechanism of BD is still unknown. Our previous study revealed the regulation of BD on gut microbiota in colitis mice. This study aimed to investigate the crosstalk between intestinal flora and host immunity in the therapeutic effect of BD on colitis.

**Methods:**

The model of colitis in mice was established using dextran sulfate sodium in drinking water, and the treatment group received BD, 5-ASA, or indole-3-propionic acid (IPA). The disease symptoms were documented, and assessments were conducted on both local and systemic inflammation as well as intestinal barrier function. The gut microbiota structure was analyzed using 16S ribosomal RNA sequencing. The metabolomic assay was performed using ultra-high performance liquid chromatography and quadrupole time-of-flight mass spectrometry, and RNA-sequencing was used to explore the mechanism of IPA on colitis treatment.

**Results:**

BD could improve colitis mice’s colonic injury and rebalance the gut microbiota dysbiosis. Fecal microbiota transplantation experiments confirmed that the therapeutic effects of BD depend on the intestinal flora, while antibiotic treatment abrogated the effect of BD. The concentration of IPA, a microbial tryptophan metabolite, was upregulated after BD-treated. IPA was further evaluated for its effect on the development of colitis and it was identified as an inhibitor of necroptosis of intestinal epithelial cells.

**Conclusions:**

Our findings suggest that BD could alleviate colitis by regulating the gut microbiota-metabolism homeostasis to inhibit the necroptosis of intestinal epithelial cells.

**Supplementary Information:**

The online version contains supplementary material available at 10.1186/s13020-025-01143-9.

## Introduction

There are two main kinds of inflammatory bowel disease (IBD), Crohn’s disease (CD) and ulcerative colitis (UC), with the characteristics of recurrence [1]. UC is an intestinal disorder that is limited to the colon. Though great effects have been done, the clear pathology of UC is still covered. Environmental factors or genes may play a vital role in the pathological mechanism of UC [2]. Commonly, UC is regarded as a typical immune disorder disease. With the development of sequencing technology, the role of gut microbiota in colonic homeostasis and pathogenesis has been confirmed according to clinical and animal studies [3–5]. A balanced intestinal flora is characterized by the dominance of obligate anaerobic members, whereas an expansion of facultative anaerobic Enterobacteriaceae is a common marker of gut dysbiosis [6]. To date, it is known that UC is a typical disorder characterized by dysbiosis and immunological disorders [7]. The crosstalk between gut microbiota and the host immune system has attracted attention, currently. The application of fecal microbiota transplantation (FMT) in the clinical practice of UC has been proven effective through short-chain fatty acids to inhibit inflammation vis suppressing histone deacetylase [8–10]. Re-building the intestinal ecosystem to rebalance the host immunity is a promising method for UC management [11, 12].

Numerous studies have demonstrated that traditional Chinese medicine serves as a regulator of intestinal flora, which can be effectively utilized in the treatment of colitis [13]. Baitouweng decoction (BD) is an ancient and effectual formula for colitis. It is composed of four herbs, *Pulsatilla chinensis *(*Bunge*)* Regel* (Baitouweng in Chinese), *Coptis chinensis Franch* (Huanglian in Chinese), *Phellodendron chinense C.K. Schneid* (Huangbai in Chinese), and *Fraxinus Chinese Roxb* (Qinpi in Chinese). In the past few decades, more and more clinical research has shown that it could effectively alleviate the syndromes of UC, such as bloody stool and abdominal pain [14–16]. Other clinical studies have reported that BD could downregulate the expression of pro-inflammatory cytokine in patients with UC [17, 18]. Previously, some studies have shown that BD could alleviate DSS-induced colitis in mice by regulating the immunity system to restore the intestinal epithelial barrier [19, 20]. Our group and other groups have found that gut microbiota may play an important role in the therapeutic effect of BD on colitis [21, 22]. Unfortunately, all of these studies have only focus on one aspect of the host or the ecological community of the intestine. The overall effect of BD on the host and microbiota has not been elucidated yet. To deeply demonstrate the co-relationship between the gut microbiota and host immunity in the effect of BD against colitis, a DSS-induced acute colitis model, gut microbiota elimination, and fecal microbiota transplantation were performed. 16S ribosomal RNA (rRNA) analysis, untargeted metabolomic, and RNA sequencing were used to identify the potential biomarkers and the underlying mechanism. Particularly, we found it is a microbiota-dependent regulation of BD on colitis, and microbiota-derived indole-3-propionic acid (IPA) plays a vital role in the effect of BD on inhibiting epithelial necroptosis.

## Materials and methods

### Drugs, chemicals and reagents

DSS (36,000–50,000 Da) was obtained from MP Biochemicals (Santa Ana, USA). Fluorescein isothiocyanate (FITC)-dextran (#46944), 5-aminosalicylic acid (5-ASA, #A79809) was purchased from Sigma-Aldrich (St. Louis, USA). Enzyme-linked immunosorbent assay (ELISA) kits for mouse IL-1β (#EK201B), IL-6 (#EK206), TNF-α (#EK282), MPO (#EK2133), and CXCL1 (#EK296) were purchased from MULTI SCIENCES (Hangzhou, China). Mouse monoclonal antibody GAPDH (#60004-1-Ig), HRP-conjugated secondary antibodies (#B900210 and #B900120), rabbit antibody RIPK3 (#17563-1-AP), mouse antibody MLKL (#66675-1-Ig), and mouse antibody phospho-RIPK1 (#66854-1-Ig) were obtained from Proteintech (Wuhan, China). Rabbit antibody Occludin (#91131), rabbit antibody phosphor-MLKL (#37333), and rabbit antibody phosphor-RIPK3 (#56532) were obtained from Cell Signaling Technology (Danvers, MA, USA). Rabbit antibody MUC2 (#ab272692), and rabbit antibody Claudin 4 (#ab210796), were pushed from Abcam (Cambridge, UK). Rabbit antibody RIPK1 (#AF7896) was purchased from Beyontime Biotechnology (Shanghai, China).

### Preparation for BD

BD is composed of four herbs which are shown in Table 1. The four herbs were combined as the ratio and submerged in distilled water (w: v, 1:10) for 30 min. Subsequently, the mixture was extracted for 1 h at 100 °C and the extraction was collected. Adding another distilled water (w: v, 1:10) for twice extraction at the same condition. Then the first and second extractions were mixed, centrifuging the mixture for 10 min at 4 °C 4000*g*. Supernatants were collected and evaporated under reduced pressure. The final concentration was 0.6825 and 0.3412 g/mL of crude drug. The administration dosage was determined according to the clinically used dosage and calculated by the body area rate of human to mouse.

**Table 1:** The comopsition of Baitouweng Decoction

Chinese name	Latin name	Part(s) used	Amount (g)
Baitouweng	*Pulsatilla* *chinensis* (*Bunge*) *Regel*	Roots	15
Huanglian	*Coptis* *chinensis* *Franch*	Roots	6
Huangbai	*Phellodendron* *chinense* *C.K. Schneid*	Roots	12
Qinpi	*Fraxinus Chinese Roxb*	Barks	12

### Quality control of BD

The extraction of BD (200 μL) was transferred to a tube and 200 μL of ice-cold methanol solution was followed by voting for 30 s and ultrasonic for 5 min. The samples were centrifuged at 12,000 rpm for 15 min at 4 °C, and the 200 μL of supernatant was used for ultra-high performance liquid chromatography and quadrupole time-of-flight mass spectrometry (UHPLC-Q-TOF-MS) analysis. The method of analysis was performed as previously described [23]. The secondary mass spectrometry database provided by Shanghai BIOTREE Biotech Co., Ltd was used to identify the active compound.

### Animals and experimental design

Male C57BL/6J mice (6–8 weeks, 20–22 g) were purchased from Vital River Laboratory Animal Technology Co., Ltd (Hangzhou, China). After acclimatization for 1 week, the mice were divided into five groups: Ctrl (n = 7), DSS (n = 8), DSS/BD-L (BD, 3.38 g/kg, crud herb, n = 8), DSS/BD-H (BD, 6.75 g/kg, crud herb, n = 8), and DSS/5-ASA (5-ASA, 100 mg/kg, n = 8). The dose for BD treatment was designed based on the clinical dosage and our previous study. The method of colitis induced was the same as previously described with a little modification [23], mice were given 3.5% DSS in drinking water for 7 consecutive days, and the drinking water was changed to distilled water from day 8 to day 14. Mice in the DSS/5-ASA, DSS/BD-L, and DSS/BD-H groups were given 5-ASA or different doses of BD, while mice in the Ctrl and DSS groups were given the same volume of distilled water. As for the Ctrl group, all mice were given distilled water for drinking during the experiment. The weight of the mice was recorded every day, and all animals were sacrificed on day 15.

To evaluate the role of gut microbiota on the protective effect of BD, pseudo-germ-free (PGF) colitis mice were established according to the previous report [24]. The mice were divided into three groups: (1) PGF-Ctrl group (n = 5), (2) PGF-DSS group (n = 8), and (3) PGF-BD group (n = 8). Briefly, all mice were treated with an antibiotic cocktail comprised of sodium ampicillin (0.5 g/L), neomycin sulfate (0.5 g/L), metronidazole (0.5 g/L), and vancomycin hydrochloride (0.25 g/L) in their drinking water during the experiment. On day 8, the mice of the PGF-DSS group and the PGF-BD group were given distilled water or BD (6.75 g/kg) combined with 3.5% DSS for the following 7 days. The body weight of the mice was recorded at the same time every day.

A fecal microbiota transplant was conducted according to the previous protocol with little modification [24]. Briefly, the mice were divided into two groups: DSS and DSS/BD group (n = 8). The gut microbiota was depleted using antibiotic cocktails (drinking water containing 0.2 g/L neomycin, metronidazole, ampicillin, and 0.1 g/L vancomycin) for 2 weeks. Then, the mice of the DSS group and DSS/BD group were orally gavaged with stool suspensions from the vehicle and BD-treated mice respectively for 4 days. After that, the mice were given 3.5% DSS for 7 days. To prepare for stool transplant, another two groups of mice (n = 5) were assigned, (1) Ctrl group: the mice were orally given distilled water, and (2) BD group: the mice were orally given BD (6.75 g/kg). After 2 weeks, the stool samples for the two groups were collected respectively every day. It was suspended with sterile pre-reduced PBS at a ratio of 10% (wt/vol) in anaerobic conditions. Then, the suspension was administrated to the recipient mice at 200 μL per mouse.

To evaluate the effect of IPA on colitis, the mice were randomly assigned into four groups: Ctrl group (Ctrl, n = 6), IPA group (IPA, n = 6), DSS group (DSS, n = 8), DSS/IPA group (DSS/IPA, n = 8). 2.5% DSS was given through drinking water for 7 days. IPA was orally given to mice at 50 mg/kg from day 1 to day 10, the mice in the Ctrl and DSS groups received equal volumes of distilled water.

### Disease activity index (DAI)

The DAI was assessed based on loss of body weight, stool consistency, and rectal bleeding. The detail was listed in the Supplementary materials (Table S1).

### Colonic tissue collected and histological analysis

Mice were anesthetized with isoflurane, the colon of mice was harvested, and the colon length was measured and recorded. About 1 cm from the distal colon was gotten and fixed in 10% (wt/vol) formalin for H&E staining and alcian blue (AB)/periodic acid (PAS) staining by using commercial kits. The images of samples were collected using a light microscope (Leica DM500). The histological damage was observed under a microscope in a blinded manner. Histological analysis was scored according to a previous report [25].

### Immunohistochemistry

After deparaffinization and hydration, the samples were blocked with 5% bovine serum albumin for 30 min followed by three times washes in PBS. Then the samples were incubated with primary antibody ZO-1 (Servicebio, GB111402) for 12 h at 4 °C. After three times washes in PBS, the slides were incubated with secondary antibody for 2 h at 37 °C. The images were collected using a light microscope (Leica DM500). The positive cells were counted using ImageJ software for optical density analysis.

### Scanning electron microscopy

About 5-mm segment of colonic tissues were immersion-fixed in 2.5% glutaraldehyde at 4 °C for 4 h. Following three sequential PBS rinses, samples underwent secondary fixation in 1% osmium tetroxide for 1 h. Subsequent processing included graded ethanol dehydration and critical-point-dried using CO_2_. Images were acquired by a transmission HT7700 electron microscope (Hitachi, Tokyo, Japan).

### Measurement of intestinal permeability

Intestinal permeability was evaluated using a FITC-dextran as described. Briefly, mice were fasted for 12 h and orally gavaged with FITC-dextran (50 mg/100 g body weight). At 4 h later, blood was collected from the facial vein. Plasma was separated by centrifugation at 6000*g* for 10 min at 4 °C, and the serum was prepared for fluorescence measurements (excitation, 490 nm; emission, 520 nm).

### Quantitative real-time PCR

Total RNA from colonic tissue was extracted using the FreeZol reagent (Vazyme, China) according to the manufacturer’s instructions. The RNA concentration was quantified using NanoDrop. The cDNA was obtained after the reverse transcription of 500 ng of RNA with a PrimerScript RT reagent Kit (Vazyme, China). Real-time PCR was performed using a power SYBR green PCR master mix (Vazyme, China) on a Roche LightCycler96 instrument. The relative gene expression was normalized to β-actin by the 2^−ΔΔCt^ method. The sequences of PCR premiers used in this study are shown in Table S2.

### Western blotting

Colonic tissue samples were homogenized in RIPA Lysis Buffer containing protease inhibitor cocktail. About 60 μg of proteins were separated by sodium dodecyl sulfate–polyacrylamide gel (SDS-PAGE). Then it was transferred to polyvinylidene fluoride (PVDF) membranes. Membranes were blocked with 5% non-fat milk (wt/vol, TBST) for 1 h at 37 °C, and probed with primary antibodies overnight at 4 °C, followed by HRP-conjugated secondary antibodies. Protein bands were developed using the ChemiDocTM XRS^+^ system (BioRad). The results were normalized to those for GAPDH.

### Cytokine analysis

Serum or colonic tissue samples were used for analysis. Colonic tissue homogenates were made in PBS followed by centrifuging at 12,000*g* for 15 min (4 °C). The suspension was collected. The amount of MPO, IL-6, TNF-α, and CXCL1 was determined by ELISA kits according to the manufacturer’s instructions. The results obtained from colonic tissue samples were normalized relative to the protein content.

### 16S rRNA gene sequencing and data analysis

The cecal contents of mice were used to extract the microbial DNA using an E.Z.N.A DNA kit (Omega Bio-Tek, Norcross, GA, U.S.). The quality of DNA was determined by agarose gel electrophoresis. The V3–V4 regions of the bacteria were amplified by polymerase chain reaction (PCR) using the primers: 338 F (5′-ACTCCTACGGGAGGCAGCAG-3′), 806R (5′-GGACTACHVGGGTWTCTAAT-3′). Then the PCR product was extracted from 2% agarose gels. After purification, the DNA amplicons were paired and sequenced on an Illumina MiSeq PE300 platform according to the standard protocols.

Operational taxonomic units (OTUs) were picked at a 97% similarity cutoff using UPARSE. Principal coordinates analysis (PCoA) was performed to visually evaluate the gut microbial difference. Bugbase was performed to predict the function of bacteria.

### Metabolomic analysis of colon tissue

The untargeted metabolomics analysis was performed as in previous reports. Approximately 10 mg tissue aliquots underwent mechanical homogenization in 25 μL ultrapure water (3 min, 4 °C), followed by metabolite extraction via 120 μL methanol supplemented with isotope-labeled internal standards. After 20-min centrifugation at 18,000×*g* (4 °C), the 20 μL supernatant was transferred to 96-well plates and mixed with 20 μL derivatization reagents.

The chemical derivatization process proceeded at 30 °C for 60 min under plate-sealed conditions. Then, Reaction mixtures were diluted with 330 μL ice-cold 50% methanol aqueous solution. Plates underwent 20-min incubation at − 20 °C prior to 30-min centrifugation at 4000×*g* (4 °C). Subsequently, 135 μL supernatants were combined with 10 μL quality control standards in new microplates, while adjacent wells received serially diluted derivatized calibration standards. The plate was sealed for analysis.

Quantitative metabolite profiling was conducted using an ACQUITY UPLC-Xevo TQ-S system (Waters Corp., USA) with ultra-performance liquid chromatography-tandem mass spectrometry (UPLC-MS/MS) detection. Analytes were resolved on a BEH C18 column system (VanGuard precolumn: 2.1 × 5 mm; analytical column: 2.1 × 100 mm, 1.7 μm) with mobile phase comprising 0.1% formic acid aqueous solution and acetonitrile (70:30 v/v) at 0.40 mL/min flow rate. Ionization parameters included 150 °C source temperature and 550 °C desolvation temperature under 1,000 L/h nitrogen gas flow. Finilary, the metabolic profile of the product was obtained.

The raw data were processed through TMBQ software (v1.0, Metabo-Profile) for peak alignment, calibration curve construction, and metabolite quantification. Principal component analysis (PCA), partial least square discriminant analysis (PLS-DA), and pathway analysis were implemented via iMAP platform (v1.0, Metabo-Profile) incorporating R-based statistical algorithms (CRAN repository). Spearman correlation analysis was used to evulate the microbiota-metabolite correlation.

### RNA-sequencing analysis of colonic tissue

Total RNA was isolated from colonic specimens using Trizol reagent (Invitrogen). Library preparation and sequencing services were conducted through LC-BIO Biotechnology Co. (Hangzhou, China). Sequencing runs were executed on an Illumina NovaSeq 6000 system, generating 2 × 150 bp paired-end reads. Bioinformatic analyses were conducted using OmicStudio cloud computing resources.

### Statistical analysis

Experimental data were analyzed in GraphPad Prism 8.0 software. One-way ANOVA was used for multiple group comparisons unless otherwise indicated. Continuous variable data were presented as mean ± SEM. The statistical significance was using asterisks as follows: * *P* < 0.05, ** *P* < 0.01, *** *P* < 0.001, and **** *P* < 0.0001.

## Results

### Quality control of BD

The UHPLC-Q-TOF-MS fingerprint of BD was performed in the total ion chromatograms (TIC) and representative compounds by the positive ESI+ (Fig. S1A) or negative ESI− mode (Fig. S1B). Components in BD were identified and the extracted ion chromatography (EIC) results are shown in Table S3.

### BD ameliorates DSS-induced colitis

**Fig. 1:**
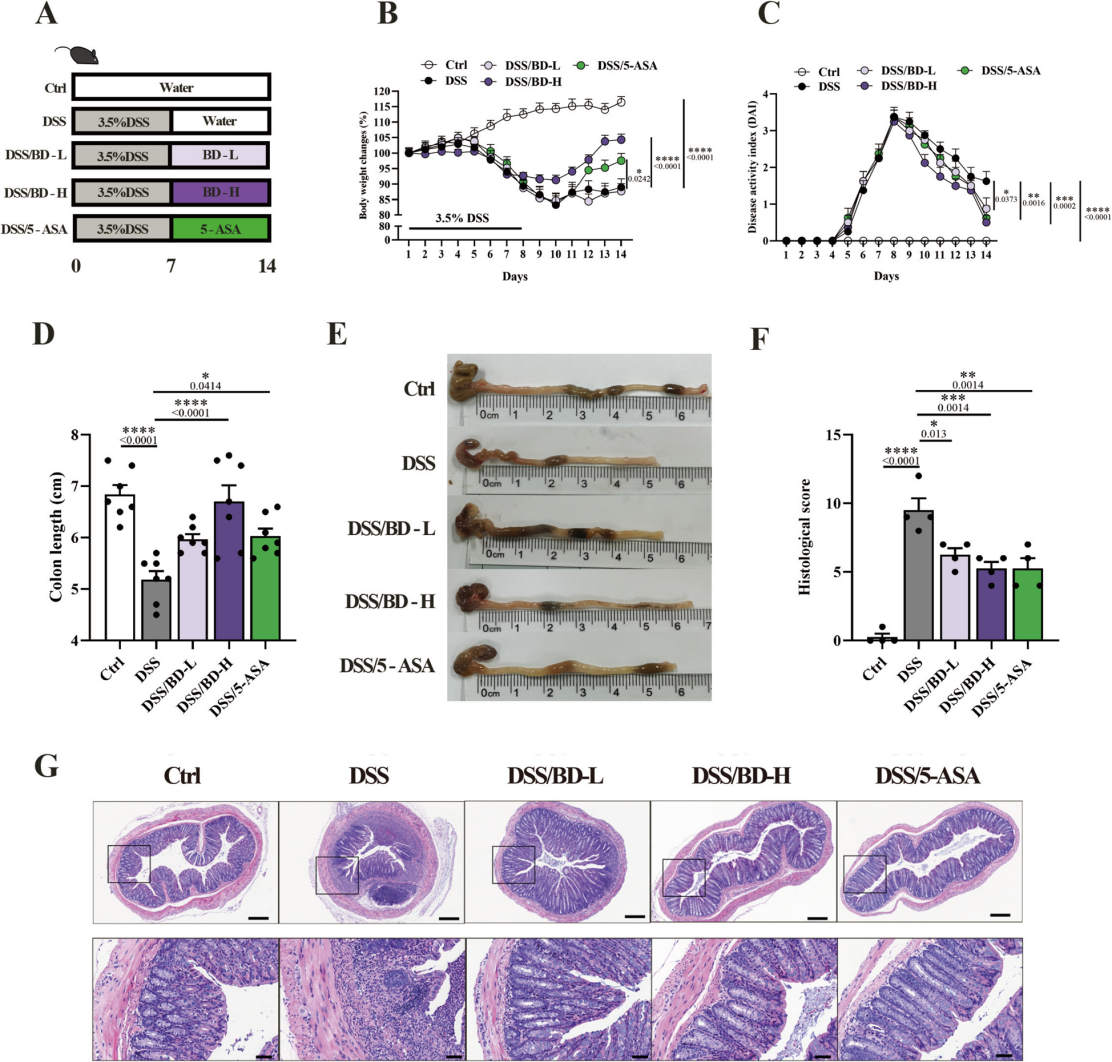
BD ameliorates DSS-induced colitis. **A** Schematic illustration of the experimental design. Mice received 3.5% DSS in drinking water for 7 days to induce colitis. From day 8 to day 14, BD treatment groups were orally administered with BD (6.75 g/kg or 3.38 g/kg), and 5-ASA treatment group was orally administered with 5-ASA (100 mg/kg) once daily. **B** Body weight change of mice during colitis progression. The weight change was expressed as a percent of the initial body weight (n = 7–8). **C** Disease activity index of mice during the course of colitis (n = 7–8). **D** Colon length of mice (n = 7). **E** Representative images of mice colon. **F** Histological score of each group (n = 4). **G** Representative H&E staining images of the colon. Scale bar: 200 μm or 50 μm. Data are expressed as Mean ± SEM. * *p* < 0.05, ** *p* < 0.01, *** *p* < 0.001, **** *p* < 0.0001; one-way ANOVA with Tukey’s post hoc analysis

Colitis was established in mice after giving 3.5% DSS in drinking water for 7 days, and mice with colitis were treated with different doses of BD or 5-ASA (Fig. 1A). As the results shown in Fig. 1B, D, and E the symptoms of colitis, such as body weight loss, and shortage of colon, were reversed after given high dose of BD and 5-ASA. The high DAI score was decreased after receiving BD or 5-ASA treatment (Fig. 1C). Histological damage, characterized by infiltration of inflammatory cells and loss of crypt in colon tissue, was a typical feature of colitis, and the H&E-staining in each group showed that BD has a protective effect on colonic structure with the lower histological score (Fig. 1F, G). Our results confirmed the therapeutical effect of BD on colitis.

### BD relieves the inflammation in colitis mice

**Fig. 2:**
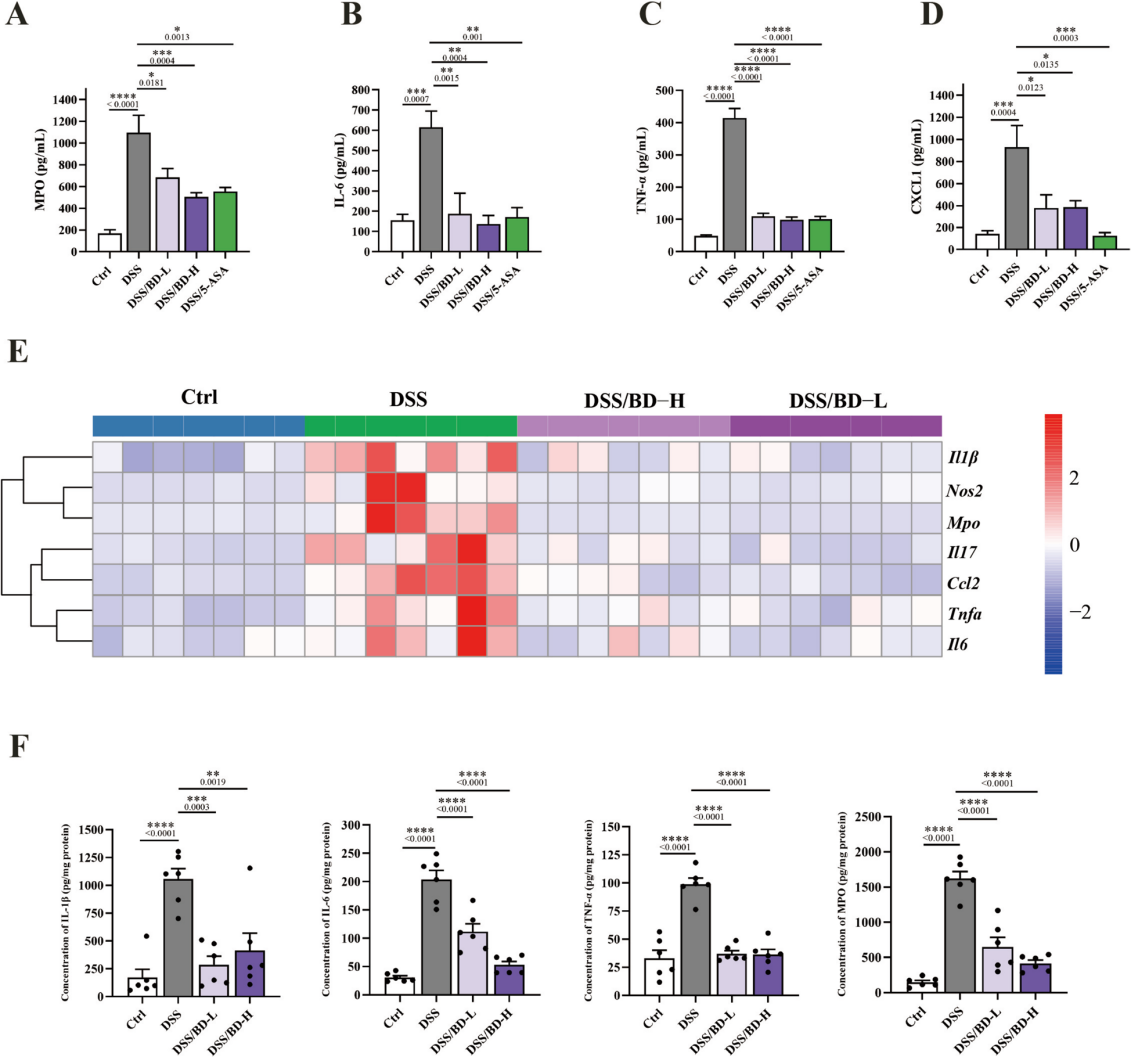
BD relieves the inflammation in colitis mice. **A**–**D** Concentration of MPO (**A**), IL-6 (**B**), TNF-α (**C**), and CXCL1 (**D**) in the serum (n = 5). **E** The heatmap of the relative mRNA expression analysis of the inflammatory cytokines *Il1β*, *Il6*, *Nos2*, *Il17*, *Ccl2*, *Tnfa*, and *Mpo* in the colon tissue of mice. *β-actin* was used for normalization and the value is expressed as fold changes compared to the Ctrl group (n = 7). **F** Concentration of IL-1β, IL-6, TNF-α, and MPO in the colonic tissue mice (n=6). Data are expressed as Mean ± SEM. * *p* < 0.05, ** *p* < 0.01, *** *p* < 0.001, **** *p* < 0.0001; one-way ANOVA with Tukey’s post hoc analysis

It is well known that colitis is a systemic inflammatory disorder. To evaluate the anti-inflammatory effect of BD, the content of MPO, IL-6, TNF-α, and CXCL1 in the serum of colitis mice were detected using ELISA, and the level of these proinflammatory cytokines was reversed after administrating BD (Fig. 2A–D). Colonic inflammation is another characteristic of colitis with significant upregulation of the proinflammatory markers. As shown in Fig. 2E, the increased mRNA levels of *Il1β*, *Il6*, *Il17*, *Tnfα*, *Mpo*, *Nos2*, and *Ccl2* were observed in the DSS group, compared with the normal mice. Giving BD could downregulate the higher level of these markers. The protein level of IL-1β, IL-6, TNF-α, and MPO in tissue was also decreased after BD-treated (Fig. 2F). Interestingly, giving the low dose of BD could not reverse the symptoms of colitis, but it could significantly inhibit the inflammation. Collectively, these results indicate that BD could prevent inflammation and oxidative stress in colitis.

### BD protects the intestinal barrier in colitis mice

**Fig. 3:**
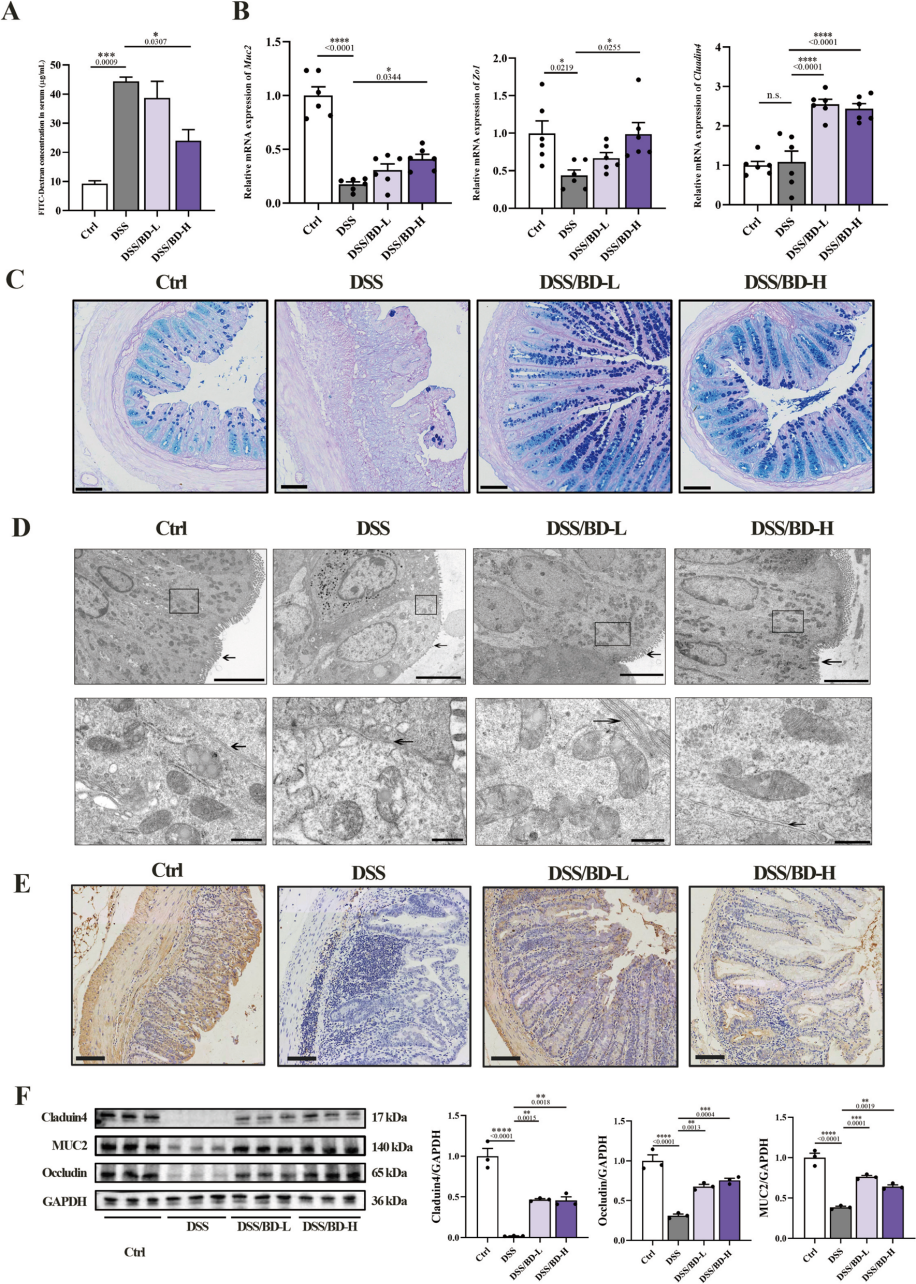
BD protects the intestinal barrier in colitis mice. **A** Intestinal leakage measured by FITC-Dextran concentration in serum (n = 3). **B** Relative mRNA expression analysis of *Muc2*, *Zo1*, and *Claduin4* in the colon tissue of mice. *β-actin* was used for normalization and the value is expressed as fold changes compared to the Ctrl group (n = 6). **C** Representative images of Alcian blue/Periodic Acid-Schiff staining and the number of goblet cells in the crypt . Scar bar: 50 μm. **D** Representative images for the microstructure of colonic epithelia by transmission electron microscope. Scar bar: 5 μm, 500 nm. **E** Immunohistochemical detection of proteins related to ZO-1. Scale bar: 50 μm. **F** Immunoblots of MUC2, Claduin4, and Occludin in the colon of mice (n = 3). Data are expressed as Mean ± SEM. * *p* < 0.05, ** *p* < 0.01, *** *p* < 0.001, **** *p* < 0.0001; one-way ANOVA with Tukey’s post hoc analysis

The damage of the intestinal barrier, which leads to increased intestinal permeability, is another feature of colitis. It is shown that greater intestinal permeability of colitis mice with a high concentration of FITC-dextran in the blood, was reversed by BD treatment (Fig. 3A). To evaluate the impact of BD on the mucosal healing of colitis, the core molecules of the intestinal barrier were evaluated. In line with this, the expression of *Muc2*, *Claduin4*, *Zo1* at mRNA level was remarkably induced in the colon of mice in the DSS group, but BD treatment significantly upregulated the expression of these markers (Fig. 3B). The decreased number of goblet cells and thickness of the mucosal barrier was significantly decreased in colitis mice, while all these changes were improved in BD-treated mice (Fig. 3C, D). Moreover, the downregulated of MUC2 and TJ protein was also observed using immunohistochemical and immunoblotting (Fig. 3E, F). This data demonstrated that BD treatment could promote mucosal healing.

### The protective effect of BD depends on the gut microbiota

**Fig. 4:**
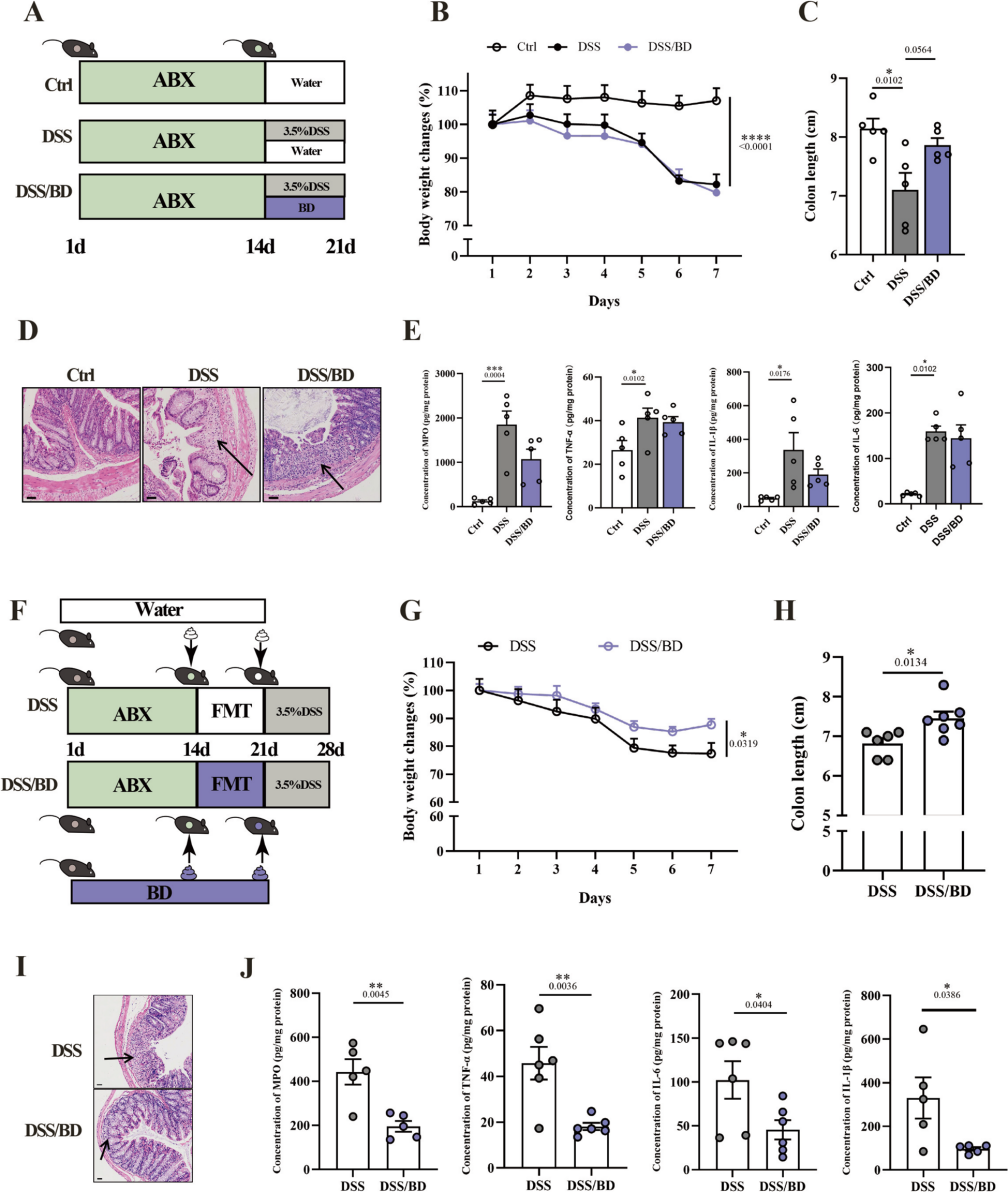
The protective effect of BD depends on the gut microbiota. **A** Schematic illustration of the experimental design of antibiotics-mediated gut microbiota depletion. Mice received DSS in drinking water for 7 days to induce colitis. BD treatment groups were orally administered with BD (6.75 g/kg) once daily from day 14 to day 21. **B** Body weight change of mice during colitis progression. The weight change was expressed as a percent of the initial body weight (n = 6–8). **C** Colon length of mice (n = 5). **D** Representative H&E staining images of the colon. Scale bar: 50 μm. **E** Concentration of IL-1β, IL-6, TNF-α, and MPO in the colon tissue (n = 5). **F** Schematic illustration of the experimental design of Fecal microbiota transplantation. **G** Body weight change of mice during colitis progression. The weight change was expressed as a percent of the initial body weight (n = 8). **H** Colon length of mice (n = 6–7). **I** Representative H&E staining images of the colon. Scale bar: 50 μm. **J** Concentration of IL-1β, IL-6, TNF-α, and MPO in the colon tissue (n = 5). Data are expressed as Mean ± SEM. * *p* < 0.05, ** *p* < 0.01, *** *p* < 0.001, **** *p* < 0.0001; one-way ANOVA with Tukey’s post hoc analysis

Gut microbiota plays a vital role in colonic health. To evaluate the role of bacteria in the pharmacodynamics of BD on colitis, microbiota-depleted mice were used (Fig. 4A). Notably, BD failed to reverse the loss of body weight and shorten colon length (Fig. 4B, C). Furthermore, the effect of protective construction disappeared (Fig. 4D) and the expression of colonic MPO, IL-1β, IL-6, or TNF-α at the protein level (Fig. 4E) was not counteracted by BD in PGF mice, indicating that the gut microbiota was essential for the effect of BD during colitis. Fecal microbiota transplantation (FMT) was performed to confirm the role of bacteria in the therapeutic effect of BD on colitis (Fig. 4F). We found that the body weight loss and colon shortening were reversed in FMT from BD-treated mice (Fig. 4G, H), as well as the histopathological damage (Fig. 4I, J). Moreover, the examination of colon cytokines showed a significant decrease in MPO, IL-6, IL-1β, and TNF-α (Fig. 4K). Taken together, these results show that gut microbiota is necessary for the protective effect of BD against colitis.

### BD counterbalances the gut microbiota of colitis

**Fig. 5:**
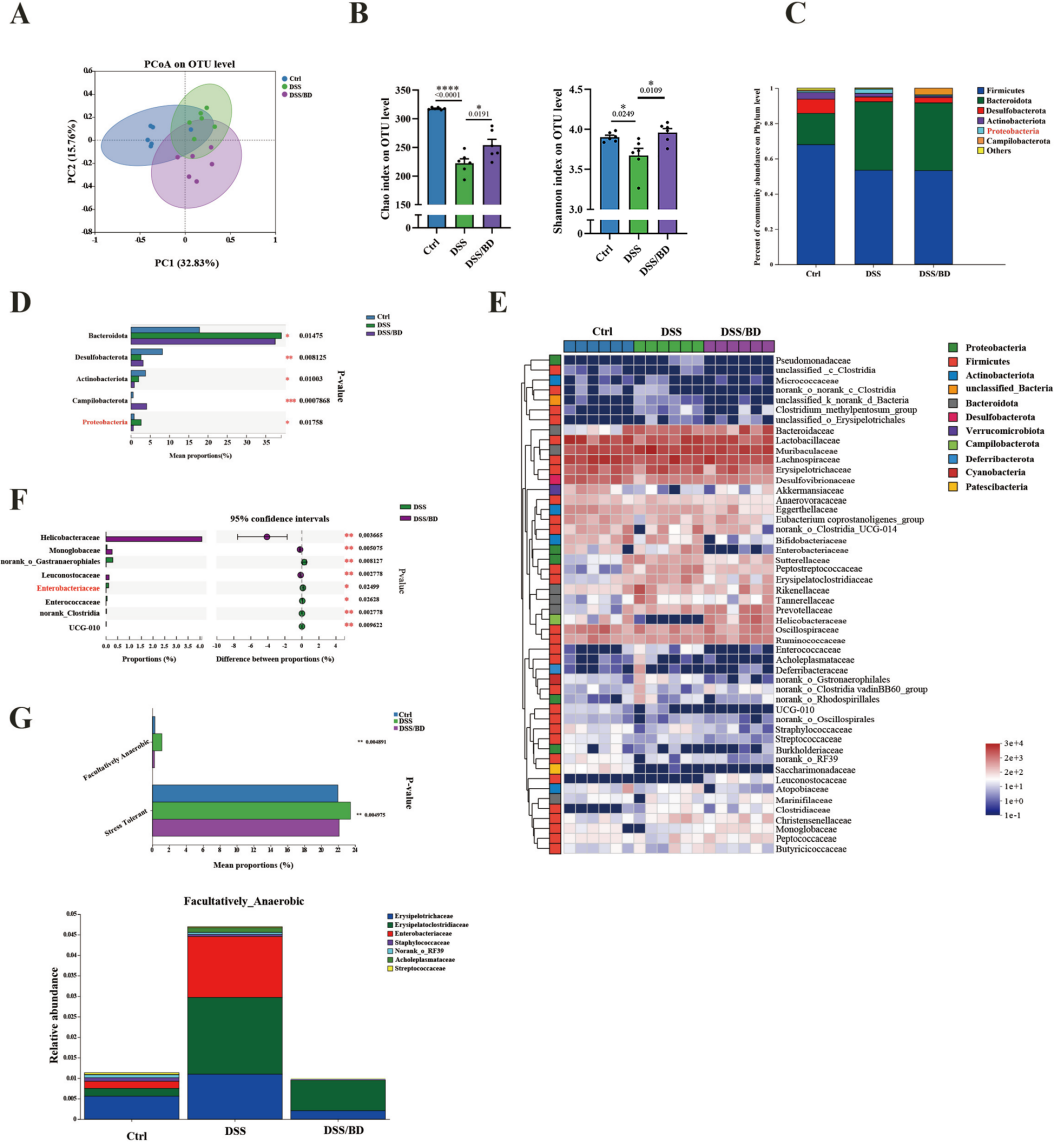
BD counterbalances the gut microbiota of colitis. **A** Principal coordinates analysis (PCoA) plotting of the gut microbiota at OTU level (n = 6). **B** Alpha diversity was evaluated from the Chao index and Shannon index (n = 6). **C** Bacterial taxonomic profiling of the colonic microbiota at the phylum level. **D** Bar plot of compositional differences at the phylum level in the gut microbiome based on the Wilcoxon rank-sum test. **E** Bacterial taxonomic profiling of the colonic microbiota at the family level. **F** Bar plot of compositional differences at the family level in the gut microbiome of mice in the DSS/BD vs. the DSS group based on the Wilcoxon rank-sum test. **G** Bar plot of compositional differences at BugBase-based phenotypic predictions, and the bar plot of the contribution for facultatively anaerobic at family level. Data are expressed as Mean ± SEM. * p < 0.05, ** p < 0.01, *** p < 0.001, **** p < 0.0001; one-way ANOVA with Tukey's post hoc analysis

To explore the impact of BD on gut microbiota, we performed high-throughput 16S rRNA gene sequencing of cecal microbiota from different groups. Using Bray–Curtis Faith-based principal coordinates analysis (PCoA) showed that the BD/DSS group displayed a shift clustering of bacterial composition, which was distinct from the DSS group (Fig. 5A), which suggested BD prevented dysbiosis. Chao and Shannon index scores are used for α-diversity analysis, consistently the results showed that BD treatment protects against the decreasing of microbiota richness and diversity (Fig. 5B). Then, we analyzed the composition of gut microbiome at phylum and family levels. BD treatment significantly reduced the relative abundance of Proteobacteria (Fig. 5C, D). The expansion of *Enterobacteriaceae*, the representative of facultatively anaerobic, was shown in the DSS group, which was significantly suppressed by BD treatment (Fig. 5E, F). The phenotypes of the gut microbiome were predicted using BugBase. The results revealed significant differences in the gene functions of facultatively anaerobic and stress-tolerant microorganisms among the three groups (Fig. 5G). Taken together, the microbial sequencing results suggest that the protective effect of BD on colitis is accompanied by a rebalanced structure of gut microbiota.

### BD treatment modulates microbial IPA production

**Fig.6:**
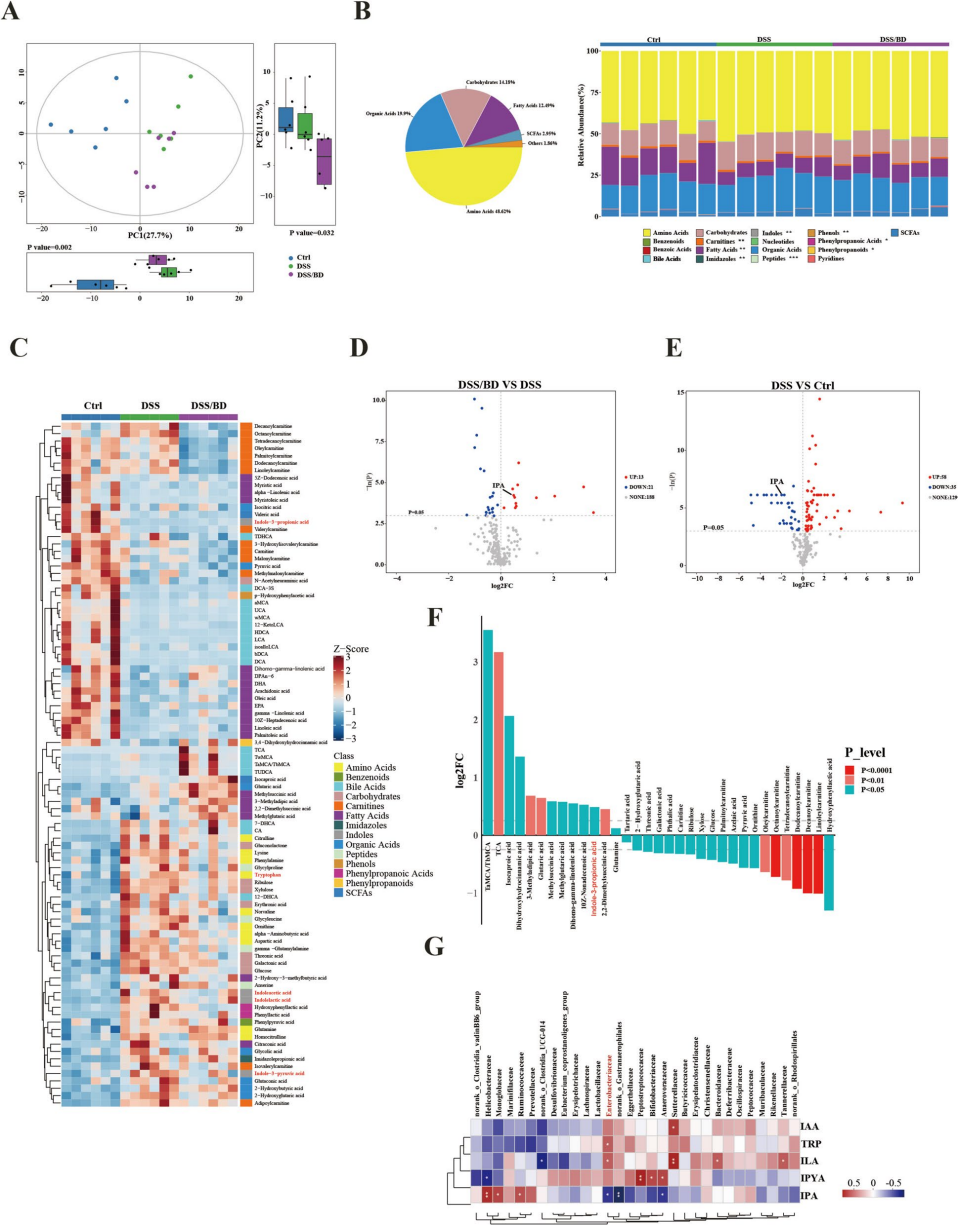
BD treatment modulates microbial IPA production. **A** The metabolomic profiles of colonic tissues were clustered using PLS-DA (n = 6). Data are presented as the mean ± SEM. P-values were determined using the nonparametric Kruskal–Wallis test. **B** Total metabolome classifications of compounds with differential metabolites in the Ctrl, DSS, and DSS/BD groups (left). The total number of significantly changed metabolites in this class is indicated, and the corresponding proportions are in parentheses (right). **C** Relative abundances of metabolites were clustered using a UPGMA dendrogram and shown in a heatmap. The color indicates the relative abundances of the metabolite in the group of samples; the corresponding relationship between the color gradient and the value is shown in the gradient color block. The metabolite variation is shown using Z_Score. **D** Volcano plot for differential metabolites in comparison between DSS/BD and DSS group. **E** Volcano plot for differential metabolites in comparison between DSS and Ctrl group. **F** Potential biomarkers of BD. **G** Correlation analysis of the association of the colitis microbes and metabolites. Data are presented as the mean ± SEM. p-values were determined using the nonparametric Kruskal–Wallis test. *p ≤ 0.05, **p ≤ 0.01, ***p ≤ 0.001. *IAA* indole-3-acetic acid, *TRP* tryptophan, *ILA* indole-lactic acid, *IPYA* indole-3-pyruvic acid, *IPA* indole-3-Propionic acid

To evaluate how BD regulates the metabolic profile of colitis, the metabolomic study of the colonic tissue of mice was performed. As expected, the metabolic profile was significantly different between the DSS group and DSS/BD group (Fig. 6A). Metabolite classification showed that 48.62% of the compound were amino acids, 19.9% were organic acids, and 14.18% were carbohydrates, while the classification of carnitines, indoles, phenols, fatty acid, imidazoles, peptides, phenylpropanoic acid, phenylpropanoids was a remarkable change among the three group (Fig. 6B). The relative quantities of colonic tissue metabolites were visualized using a heatmap, and the top 93 metabolites are listed and clustered in Fig. 6C. The differential metabolites between two groups was shown in the volcano plot (Fig. 6D, E), and BD treatment significantly restored the concentration of IPA. Potential biomarker analysis showed that IPA was one of the biomarkers between the DSS group and the DSS/BD group (Fig. 6F). Integrative analysis was performed to determine the potential association between gut microbiota and metabolites in colitis. We found that enrichment of *Helicobacteraceae*, *Monoglobaceae*, and *Ruminococcaceae* was positively correlated with IPA, while *Enterobacteriaceae* was negatively correlated with IPA (Fig. 6G). These results suggest that the therapeutic effect of BD is associated with the bacterial metabolites of IPA.

### IPA alleviates DSS-induced colitis in mice

**Fig.7:**
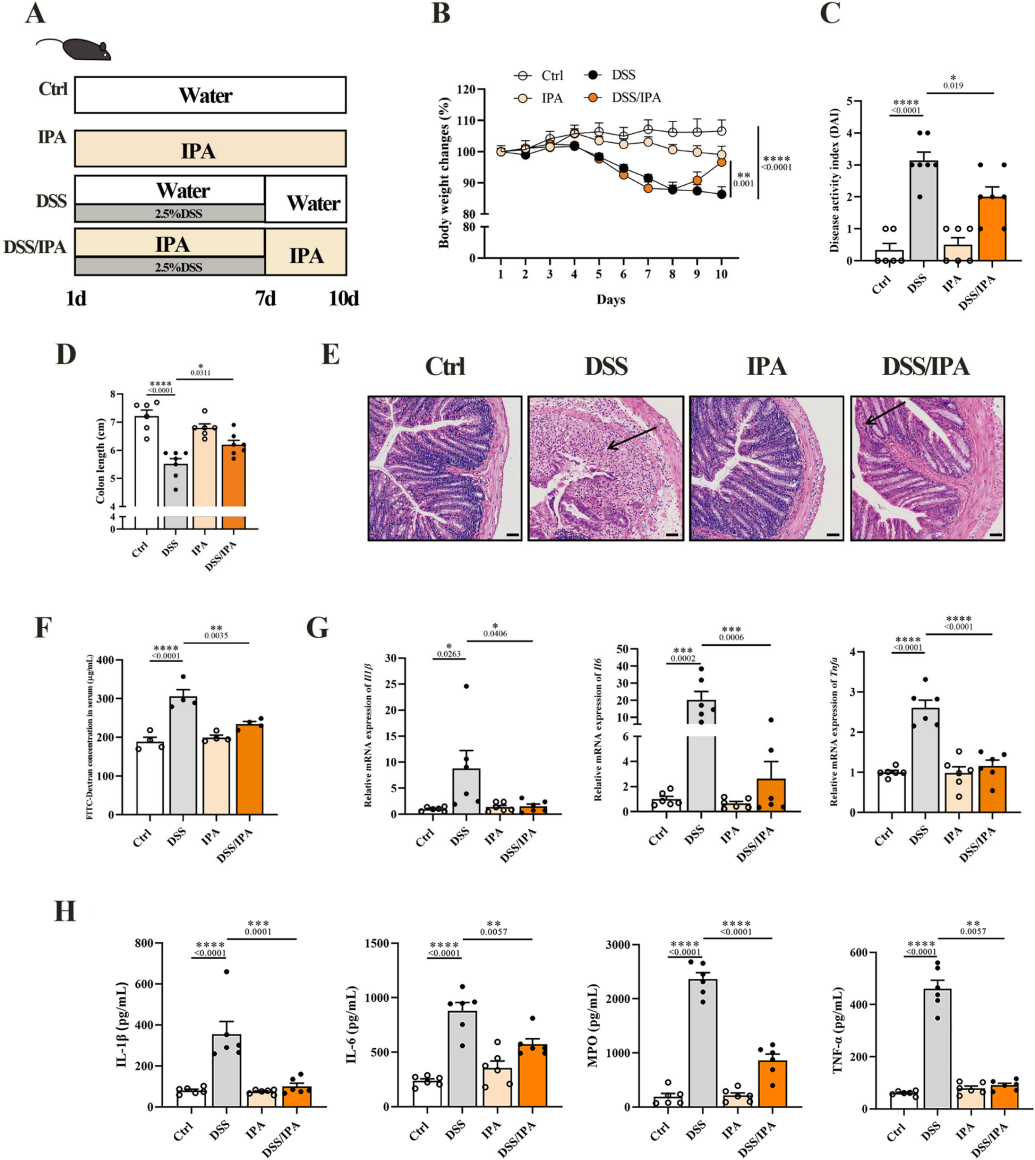
IPA alleviates DSS-induced colitis in mice. **A** Schematic illustration of the experimental design. Mice received DSS in drinking water for 7 days to induce colitis. **B** Body weight change of mice during colitis progression (n = 6–8). The weight change was expressed as a percent of the initial body weight. **C** The disease active index score on day 10 (n = 6–7). **D** Colon length of mice. **E** Representative H&E staining images of the colon . Scale bar: 50 μm. **F** Intestinal leakage was measured by FITC-Dextran concentration in serum (n = 4). **G** Relative mRNA expression analysis of *Il1β*, *Il6*, *Tnfa*, and *Mpo* in the colon tissue of mice (n = 6). *β-actin* was used for normalization and the value is expressed as fold changes compared to the Ctrl group. **H** The concentration of IL-1β, IL-6, TNF-α, and MPO in the serum (n = 6). Data are expressed as Mean ± SEM. * p < 0.05, ** p < 0.01, *** p < 0.001, **** p < 0.0001; one-way ANOVA with Tukey’s post hoc analysis

Since great attention has been focused on the role of tryptophan (Trp) in colitis, the effect of IPA on colitis is still elusive. To evaluate the role of IPA on the development of colitis, exogenous IPA was administrated to colitis mice (Fig. 7A). Significantly, the loss of body weight (Fig. 7B), DAI (Fig. 7C), and colon shortening (Fig. 7D) were reversed. The protective effects of IPA on the mucosal barrier were shown in histopathological analysis (Fig. 7E), evaluated by intestinal permeability (Fig. 7F). Moreover, the pro-inflammatory cytokines, both in mRNA level and protein level, were blunted in colitis mice receiving IPA (Fig. 7G, H). Taken together, these results indicate that IPA could significantly protect against colitis.

### IPA inhibits the necroptosis of the intestinal epithelium

**Fig. 8:**
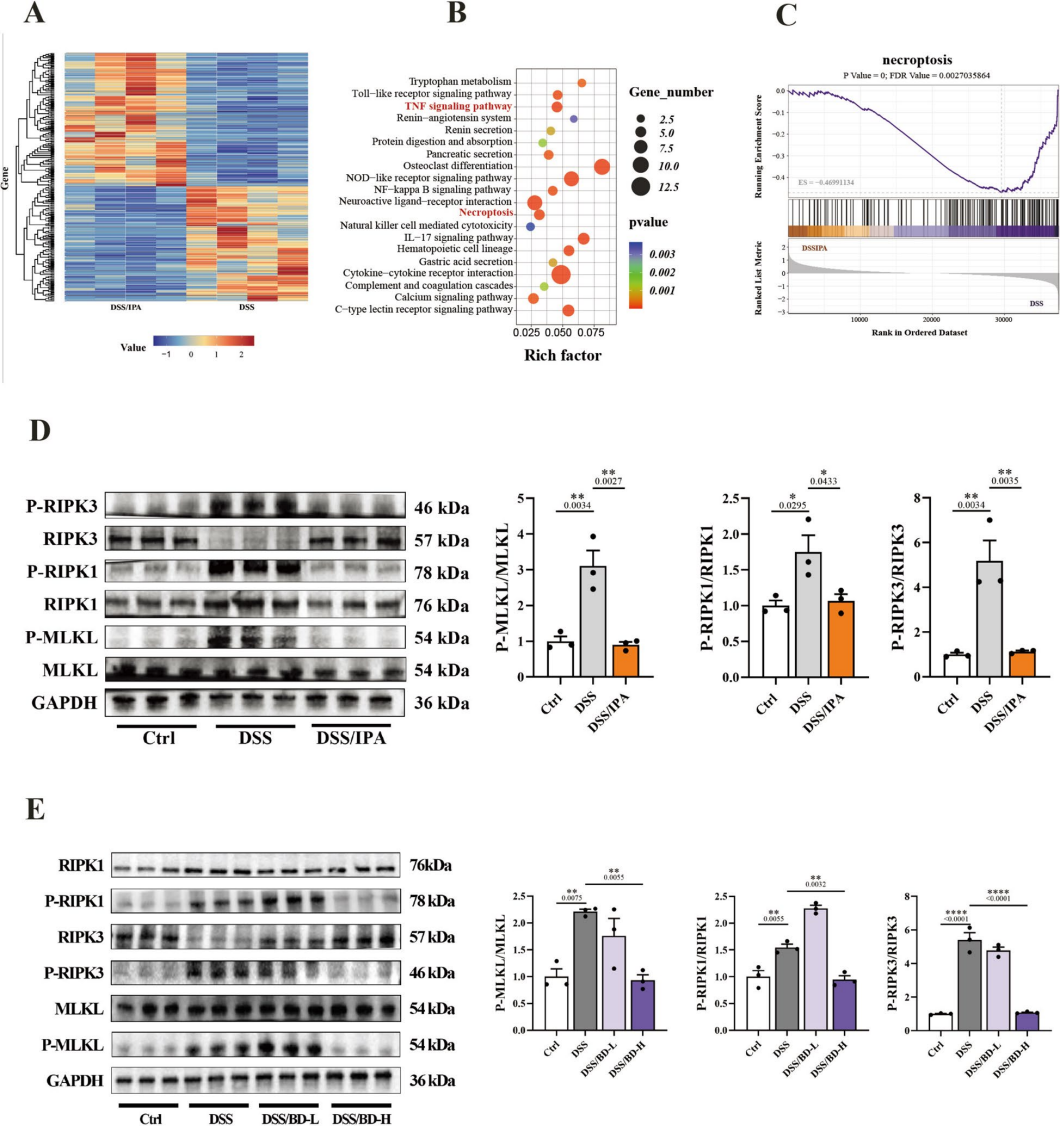
IPA inhibits the necroptosis of intestinal epithelium. **A** The heatmap of the different genes between DSS and DSS/IPA group (n = 4). **B** Kyoto Encyclopedia of Genes and Genomes (KEGG) pathway enrichment analysis of the most significantly changed pathways. **C** The GSE of necroptosis. **D** Immunoblots of necroptosis marker protein (RIPK1/3, MLKL) in the colonic tissue of mice treated with or without IPA (n = 3). **E** Immunoblots of necroptosis marker protein (RIPK1/3, MLKL) in the colonic tissue of mice treated with or without BD (n = 3). Data are expressed as Mean ± SEM. * p < 0.05, ** p < 0.01, *** p < 0.001, **** p < 0.0001; one-way ANOVA with Tukey’s post hoc analysis

To investigate the deep mechanism of IPA on reliving colitis, RNA-sequencing was performed. Of the 37605 gene transcripts expressed, about 261 genes differed significantly between the DSS group and the DSS/IPA group (Fig. 8A). Kyoto Encyclopedia of Genes and Genomes (KEGG) pathway analysis of differentially expressed genes showed enrichment in a series of signaling pathways (Fig. 8B). Of note, Necroptosis was the top 20 enriched functional pathway (Fig. 8C). Using immunoblotting, we examined the expression levels of the core protein, RIPK1/3 and MLKL, of necroptosis in the colonic tissue of mice in both IPA-treated and BD-treated mice. The findings indicated that IPA and BD treatment could remarkably decrease the level of p-RIPK1/3 and p-MLKL (Fig. 8D, E). Overall, these findings suggest microbial IPA may inhibit the epithelial necroptosis to maintain the integrity of intestinal epithelium.

## Discussion

**Fig. 9:**
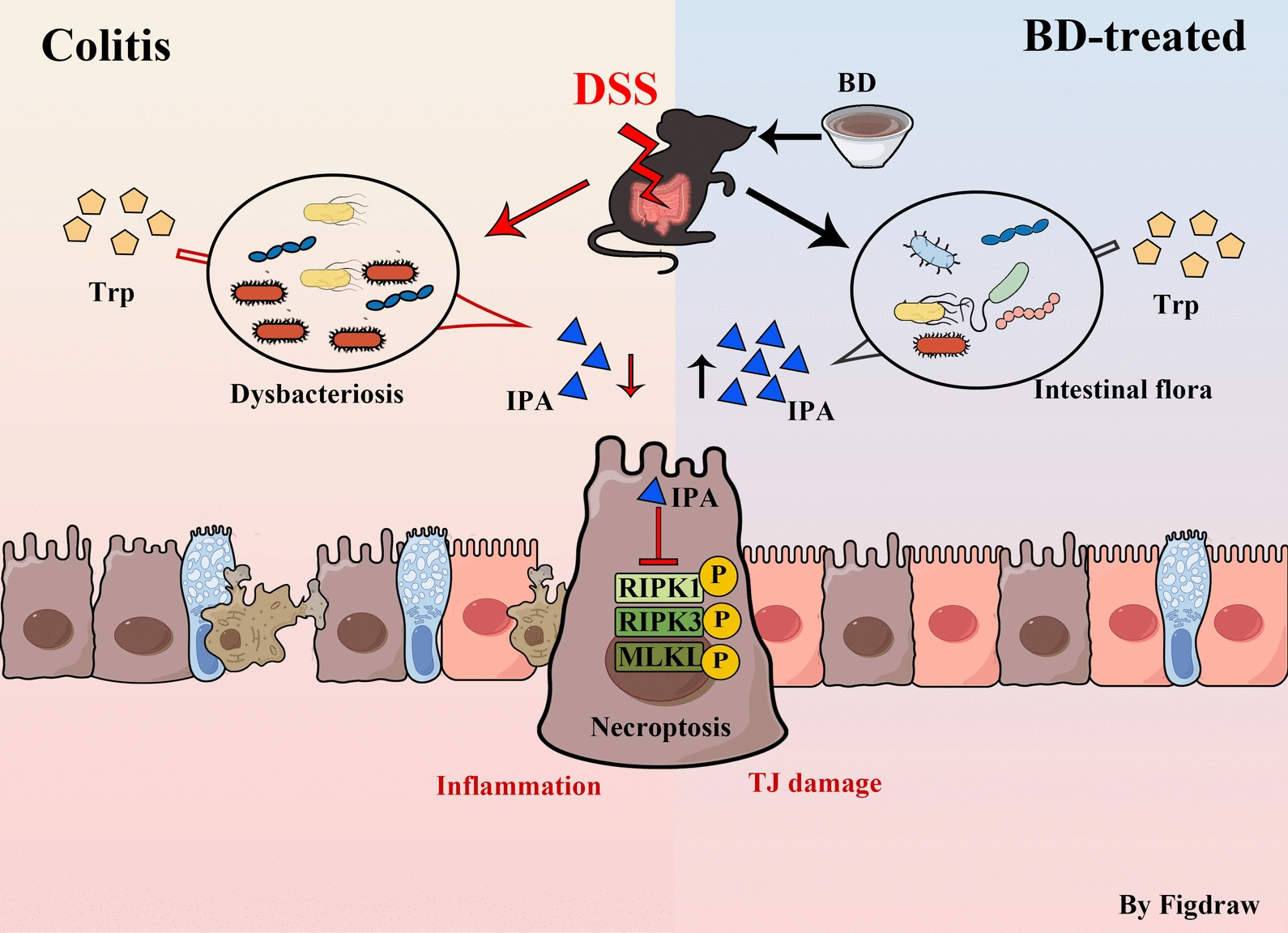
Schematic diagram of the mechanism underlying BD-alleviated colitis. *DSS* dextran sulfate sodium salt, *BD* Baitouweng Decoction, *Trp* tryptophan, *IPA* indole-3-propionic acid, *TJ* tight junction

The crosstalk between gut microbiota and host immunity is involved in the pathologies of colitis. Exploring the insight mechanism is essential for achieving treatment goals. In this study, by exploring the relationship between the immunity regulation of BD with the gut microbiota, we identified IPA, a microbial metabolite of Trp, that inhibits the necroptosis of the intestinal epithelium through RIPK1/3-MLKL pathway and promotes mucosal healing. These findings have elucidated the metabolic mechanism of BD on colitis and given us new insight into the change in the production of microbial metabolite in colitis and host immunity regulation (Fig. 9).

BD, derived from Treatise on Cold Damage (Shang-Han-Lun in Chinese), is a famous herbal formula for intestinal disorders, such as colitis and diarrhea. It has been widely used in clinical practice with well-confirmed effects for more than 2000 years. Previous studies have shown the benefits of BD on immunoregulatory effects of intestinal homeostasis, which focus on regulating Th17/Treg balance [20] and the IL-6/STAT3 pathway. Our group and other groups have found the effect of BD on regulating gut microbiota [22, 26]. However, the effect of BD on the correlation between gut microbiota and the immune process on colitis has not been clarified. Based on the previous reports, our study supports that the regulation of the gut microbiota is essential for BD acting as an immune regulator in colitis treatment. In this study, we found that the protective effect of BD against colitis was blunted after gut microbiota depletion and was transmissible by FMT.

The crosstalk between microbial metabolites and host immunity is the key regulation of host health [27]. Previously, some studies have found the effect of BD on regulating the metabolism of short-chain fatty acids (SCFAs) [22] and bile acids [26] using targeted metabolome. To evaluate the bacterial metabolites on the host immune response, we explore the change of microbial metabolites of colonic tissue. In this study, we found that the metabolism of Trp was significantly changed, as well as microbial indoles. Previously, some studies have reported that a higher concentration of indoleacetic acid (ILA), a microbial metabolite of Trp, may exacerbate colitis [24]. We similarly found that the high concentration of ILA in colonic tissue of colitis mice, however BD-treated could significantly not downregulate the level of ILA. Of note, IPA has attracted us. A lower level of IPA in the serum or colonic biopsy samples of colitis patients has been reported [28]. There is the lower level of IPA in the colonic tissue of mice, and it was remarkably increased after BD treatment. This fact makes us deeply explore the insight mechanism of the change of IPA during colitis and BD treatment.

Recent studies have shown that microbial metabolites of Trp may play different roles in intestinal immunity through the aryl hydrocarbon receptor (AhR) [29, 30]. In a previous study, the anti-inflammatory effect of IPA was confirmed in osteoarthritis via the AhR/nuclear factor kappa-B (NF-κB) axis [31]. Another investigation found that microbial IPA may restrain inflammation and fibrosis by activating the pregnane X receptor (PXR) [32]. Here we found that, using RNA-sequencing, IPA could dampen the inflammation and promote the mucosal healing of the colon, which may correlate with inhibiting the necroptosis.

Necroptosis, which is often along with apoptosis, plays a role in intestinal cell damage [33–35]. TNF-induced necroptosis activates the RIPK1 to bind to RIPK3 and generate a necrosome complex [36]. It then promotes the phosphorylation of MLKL, which oligomerizes and translocates to the plasma membrane, causing necroptotic cell death [37]. The activation of the RIPK3/MLKL pathway has been well-confirmed in human IBD [38] and murine model of colitis [39]. Pharmacologically inhibiting the necroptosis is a potential method to deal with the mucosal damage in colitis [40, 41]. Our results support that IPA, a bacterial metabolite, may block the epithelial necroptosis by inhibiting the RIPK1/RIPK3/MLKL pathway, which may give a rational explanation for the lower concentration of its in both colonic tissue and serum of colitis.

Although BD is a promising anti-UC drug, this study still has some limitations. First of all, we used the FMT to evaluate the gut microbiota on the therapeutic effect of BD in colitis, the exact composition of bacteria needs to be further studied. Secondly, we found that a decrease of IPA may have contributed to the development of colitis in mice, there was a lack of clinical validation. Finally, we conducted a qualitative analysis of the components of BD but lacked a quantitative analysis of the main components. The quantitative analysis of BD will be performed in our future study.

## Conclusion

In the present study, we reported that BD is protective against colitis mice via the gut microbiota-IPA-epithelial necroptosis axis. BD promotes the enrichment of microbiota-derived Trp metabolite IPA, which inhibits the RIPK1/RIPK3/MLKL signaling pathway to suppress the necroptosis of intestinal epithelial cells and improve barrier function. Our results offer a new insight into the benefits of BD on colitis, and highlight that IPA may be a potential new medication for colitis.

## Supplementary Information

Supplementary Material 1

Supplementary Material 2

## Data Availability

All data in this study are available from the corresponding author upon reasonable request.

## Abbreviations

IBD: inflammatory bowel disease
UC: ulcerative colitis
CD: Crohn's disease
FMT: fecal microbiota transplantation
BD: baitouweng decoction
IPA: indole-3-propionic acid
FITC: fluorescein isothiocyanate
5-ASA: 5-aminosalicylic acid
ELISA: enzyme-linked immunosorbent assay
UHPLC-Q-TOF-MS: ultra-high performance liquid chromatography and quadrupole time-of-flight mass spectrometry
PGF: pseudo-germ-free
DAI: disease activity index
AB: alcian blue
PAS: periodic acid
SDS-PAGE: sodium dodecyl sulfate-polyacrylamide gel
PVDF: polyvinylidene fluoride
OTUs: operational taxonomic units
PCoA: principal coordinates analysis
UPLC-MS/MS: ultra-performance liquid chromatography coupled to tandem mass spectrometry
PCA: principal component analysis
PLS-DA: partial least square discriminant analysis
TIC: total ion chromatograms
EIC: extracted ion chromatography
Trp: tryptophan
KEGG: Kyoto Encyclopedia of Genes and Genomes
SCFAs: short-chain fatty acids
ILA: indoleacetic acid
AhR: aryl hydrocarbon receptor
NF-κb: nuclear factor kappa-B
PXR: pregnane X receptor
