# Supplementary material for: Baitouweng decoction modulates gut microbial production of indole-3-propionic acid and epithelial necroptosis to alleviate DSS-induced colitis in mice
Source: Chin Med. 2025 Jul 31;20:119. doi: 10.1186/s13020-025-01143-9 (PMC12312286; doi:10.1186/s13020-025-01143-9)
Supplement: Supplementary file 1 — Supplementary Material 1 [file 13020_2025_1143_MOESM1_ESM.docx]

**Supplementary Information**

Table S1 DAI score

| DAI Score | Weight Loss | Stool Consistency | Gross Bleeding |
| --- | --- | --- | --- |
| 0 | 0 | Normal | Normal |
| 1 | 1-5% | - |  |
| 2 | 5-10% | Loose | Guiac (+) |
| 3 | 10-15% | - |  |
| 4 | >15% | Diarrhea | Gross Bleeding |

Table S2 mRNA primers

| **Genes** | **Primers** | **Primer Sequences (5′-3′)** |
| --- | --- | --- |
| *Mouse-Il1β* | F | 5’-AATGCCACCTTTTGACAGTGATG-3’ |
|  | R | 5’-GGAAGGTCCACGGGAAAGAC-3’ |
| *Mouse-Tnfα* | F | 5’-AAGGCCGGGGTGTCCTGGAG-3’ |
|  | R | 5’-AGGCCAGGTGGGGACAGCTC-3’ |
| *Mouse-Il6* | F | 5’-CCACTTCACAAGTCGGAGGCTTA-3’ |
|  | R | 5’-AGTGCATCATCGTTGTTCATAC-3’ |
| *Mouse-Il17* | F | 5’-CCACGTCACCCTGGACTCTC-3’ |
|  | R | 5’-CTCCGCATTGACACAGCG-3’ |
| *Mouse-Mpo* | F | 5’-GAGTCCCACTCAGCAAGGTC-3’ |
|  | R | 5’-TCTGGCGATTCAGTTTGGCT-3’ |
| *Mouse-Ccl2* | F | 5’-CCTGCTGCTACTCATTCACCA-3’ |
|  | R | 5’-ATTCCTTCTTGGGGTCAGCA-3’ |
| *Mouse-Nos2* | F | 5’-TTGGGTCTTGTTCACCACGG-3’ |
|  | R | 5’-CCTCTTTCAGGTCACTTTGGTAGG-3’ |
| *Mouse-Zo1* | F | 5’-AGTTCTGCCCTCAGCTACCA-3’ |
|  | R | 5’-GCTTAAAGCTGGCAGTGTC-3’ |
| *Mouse-Muc2* | F | 5’-TGTGTTTCAGGCTCCATCAC-3’ |
|  | R | 5’-TGCAGCCATTGTAGGAAATC-3’ |
| *Mouse-Occludin* | F | 5’-ACAAAGAGCTCTCTCGTCTCG-3’ |
|  | R | 5’-CATAGTCTCCCACCATCCTC-3’ |
| *Mouse-Claudin4* | F | 5’-GGGGATCATCCTGAGTTGTG-3’ |
|  | R | 5’-CACTGCATCTGACCTGTGCT-3’ |
| *Mouse-**β-actin* | F | 5’-CTCATGAAGATCCTGACCGAG-3’ |
|  | R | 5’-AGTCTAGAGCAACATAGCACAG-3’ |

Table S3 UHPL-Q-TOF-MS of BD

| NO. | Name | Formula | RT (min) | Mode | Intensity |
| --- | --- | --- | --- | --- | --- |
| ① | Ferulic acid | C10H10O4 | 1.74 | Positive | 94265550.12 |
| ② | Berberine | C20H18NO4+ | 3.13 | Positive | 7073984779 |
| ③ | Pulchinenoside C | C59H96O26 | 4.22 | Positive | 95922318.36 |
| ④ | Esculin | C9H6O4 | 0.94 | Negative | 2146240853 |
| ⑤ | Rosmarinic acid | C18H16O8 | 2.49 | Negative | 108792875.4 |

**Figure S1. Analysis of the components of BD by UHPLC-Q-TOF-MS.** (A) The total ion chromatograms and the chemical structure and extracting ion chromatography (EIC) of representative compounds in positive mode. (B) The total ion chromatograms and the chemical structure and extracting ion chromatography (EIC) of representative compounds in negative mode.
