# Supplementary figures and images for: Baitouweng decoction modulates gut microbial production of indole-3-propionic acid and epithelial necroptosis to alleviate DSS-induced colitis in mice
Source: Chin Med. 2025 Jul 31;20:119. doi: 10.1186/s13020-025-01143-9 (PMC12312286; doi:10.1186/s13020-025-01143-9)

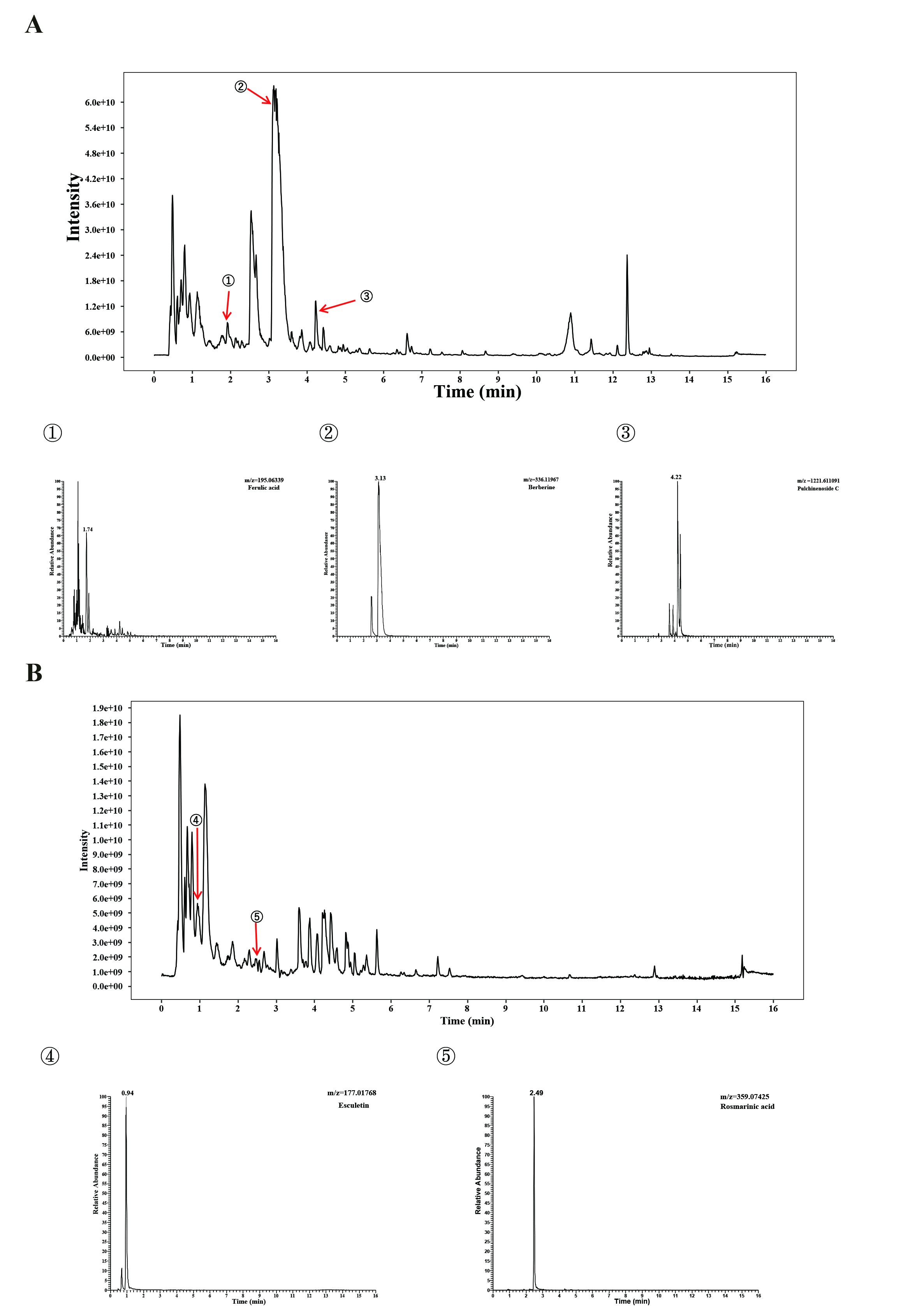

Supplement: Supplementary file 2 — Supplementary Material 2 [file 13020_2025_1143_MOESM2_ESM.tif]
